# Supplementary material for: Comprehensive analysis of total knee arthroplasty kinematics and functional recovery: Exploring full-body gait deviations in patients with knee osteoarthritis
Source: PLoS One. 2024 Dec 5;19(12):e0314991. doi: 10.1371/journal.pone.0314991 (PMC11620450; doi:10.1371/journal.pone.0314991)
Supplement: S1 Table — (DOCX) [file pone.0314991.s001.docx]

**Supporting Information 1:** **Clinical features and patient reported outcome measures of patients (median [IQR] or n patient & percentage), Clusters and Control Group.**

| **Features** | **All Patients (n=100)** | **Cluster 1**  **(n=59)** | **Cluster 2**  **(n=20)** | **Cluster 3**  **(n=21)** | **Control Group** | **KW** | **Between Clusters Comp.** | | | **Comp. with Control Group** | | |
| --- | --- | --- | --- | --- | --- | --- | --- | --- | --- | --- | --- | --- |
|  |  |  |  |  |  |  | **1 vs 2** | **2 vs 3** | **3 vs 1** | **CL1** | **CL2** | **CL3** |
| **Pre-Surgery** | | | | | | | | | | | | |
| Pain (0-10) | 3.7 [3.2] | 2.7 [3.6] | 4.7 [2.2] | 5 [2.5] | 0 [0] | < 0.01 | < 0.01 | - | < 0.01 | < 0.01 | < 0.01 | < 0.01 |
| SF12 PCS | 34.3 [9.4] | 35.6 [11.5] | 33.7 [10] | 31.5 [7.7] | 54.5 [4.9] | - | - | - | - | < 0.01 | < 0.01 | < 0.01 |
| SF12 MCS | 45.8 [18.2] | 48.3 [18.6] | 44.6 [20.5] | 38.3 [19.6] | 53.7 [7.3] | - | - | - | - | < 0.01 | 0.02 | < 0.01 |
| WOMAC P | 45 [25] | 45 [25] | 50 [25] | 40 [22.5] | 100 [6.3] | - | - | - | - | < 0.01 | < 0.01 | < 0.01 |
| WOMAC F | 46.4 [21.4] | 50 [21.4] | 51.8 [25] | 39.3 [16.1] | 100 [3.6] | - | - | - | - | < 0.01 | < 0.01 | < 0.01 |
| **One-year post-surgery** | | | | | | | | | | | | |
| Pain (0-10) | 0 [1.2] | 0 [0.6] | 0 [1.2] | 0 [2] | - | - | - | - | - | < 0.01 | < 0.01 | < 0.01 |
| SF12 PCS | 45.2 [15.7] | 45.1 [16.9] | 45.6 [11.5] | 44.7 [13.4] | - | - | - | - | - | < 0.01 | < 0.01 | < 0.01 |
| SF12 MCS | 51.3 [18.5] | 52.8 [17.7] | 53.3 [19.9] | 49.9 [17.1] | - | - | - | - | - | - | - | - |
| WOMAC P | 85 [15] | 85 [16.3] | 90 [17.5] | 85 [27.5] | - | - | - | - | - | < 0.01 | < 0.01 | < 0.01 |
| WOMAC F | 85.7 [25] | 85.7 [25.9] | 85.7 [25] | 85.7 [30.4] | - | - | - | - | - | < 0.01 | < 0.01 | < 0.01 |
| Satisfaction (%) | 77 (79%) | 47 (82%) | 14 (70%) | 16 (76%) | - | - | - | - | - | - | - | - |

*KW stands for Kruskall-Walis tests between clusters. Between clusters comp. regroups the post-hoc tests (Wilcoxon). Comp. with Control Group shows the comparison*

*between cluster before or one year after surgery with the Control Group (Wilcoxon).*
