# Supplementary material for: Comprehensive analysis of total knee arthroplasty kinematics and functional recovery: Exploring full-body gait deviations in patients with knee osteoarthritis
Source: PLoS One. 2024 Dec 5;19(12):e0314991. doi: 10.1371/journal.pone.0314991 (PMC11620450; doi:10.1371/journal.pone.0314991)
Supplement: S2 Table — (DOCX) [file pone.0314991.s002.docx]

**Supporting information 2:** **Kinematic Features (median [IQR]) before surgery for Clusters and Control Group.**

|  | **Features (deg)** | **Cluster 1**  **(n=59)** | **Cluster 2**  **(n=20)** | **Cluster 3**  **(n=21)** | **Control Group** | **KW** | **Between Clusters Comp.** | | | **Comp. with Control Group** | | |
| --- | --- | --- | --- | --- | --- | --- | --- | --- | --- | --- | --- | --- |
|  |  |  |  |  |  |  | **1 vs 2** | **2 vs 3** | **3 vs 1** | **CL1** | **CL2** | **CL3** |
| **Thorax** | | | | | | | | | | | | |
| Sagittal | TASa | 3.4 [7] | 7.6 [8.4] | 6 [8] | 2.4 [6.9] | 0.037 | 0.015 | - | - | - | < 0.01 | - |
|  | TASb | 2.7 [7.2] | 5.8 [8] | 5.1 [7.7] | 1.5 [6.9] | - | - | - | - | - | < 0.01 | - |
|  | TASc | 4.6 [6.8] | 8.4 [8.2] | 7.2 [9.4] | 3.2 [7.4] | 0.029 | 0.017 | - | - | - | < 0.01 | 0.035 |
|  | TASd | 2.4 [7.7] | 5.5 [8.1] | 4 [7.8] | 1.3 [7.2] | 0.032 | 0.013 | - | - | - | 0.01 | - |
|  | ROM | 2.7 [1.4] | 2.5 [1.6] | 4 [2.2] | 2.4 [0.9] | < 0.01 | - | < 0.01 | < 0.01 | < 0.01 | - | < 0.01 |
| Frontal | TAFa | -0.2 [2.8] | -0.6 [2.5] | 0.3 [2.4] | -0.3 [2.6] | - | - | - | - | - | - | - |
|  | TAFb | -2.6 [3.8] | -2 [4] | -3.9 [3.6] | -1.5 [3] | - | - | - | - | 0.021 | - | < 0.01 |
|  | TAFc | 1.3 [3.8] | 1.6 [1.9] | 1.9 [2.4] | 1.6 [2.8] | - | - | - | - | - | - | - |
|  | ROM | 4.2 [2.7] | 3.5 [2.7] | 5.2 [4.6] | 2.6 [2.2] | - | - | - | - | < 0.01 | - | < 0.01 |
| Transverse | TATa | -2.1 [4.2] | -3 [3.4] | -2.1 [3.1] | -1.9 [4.1] | - | - | - | - | - | - | - |
|  | TATb | 0.8 [4] | 1.6 [3.3] | 1 [4.6] | 2.1 [4.6] | - | - | - | - | - | - | - |
|  | ROM | 5.4 [3.6] | 5.4 [3.2] | 6.3 [2.4] | 5.5 [4.3] | - | - | - | - | - | - | - |
| Pelvis | | | | | | | | | | | | |
| Sagittal | PASa | 14.1 [5.4] | 4.5 [4.7] | 8.5 [3.4] | 6.7 [5.7] | < 0.01 | < 0.01 | < 0.01 | < 0.01 | < 0.01 | < 0.01 | - |
|  | PASb | 13.2 [4.9] | 3.2 [3.7] | 8.2 [4] | 6.4 [6.1] | < 0.01 | < 0.01 | < 0.01 | < 0.01 | < 0.01 | < 0.01 | - |
|  | PASc | 15.2 [3.9] | 5.9 [4.2] | 11 [5.2] | 7.9 [6] | < 0.01 | < 0.01 | < 0.01 | < 0.01 | < 0.01 | < 0.01 | < 0.01 |
|  | PASd | 12.4 [4.6] | 3.5 [4.1] | 7.6 [4.1] | 6 [6.2] | < 0.01 | < 0.01 | < 0.01 | < 0.01 | < 0.01 | < 0.01 | - |
|  | ROM | 2.9 [1.7] | 2.8 [1.5] | 3.5 [2.3] | 2.2 [1.1] | - | - | - | - | < 0.01 | - | < 0.01 |
| Frontal | PAFa | -0.2 [2.9] | -0.5 [3.1] | 0.2 [3.7] | -0.1 [2.1] | - | - | - | - | - | - | - |
|  | PAFb | 2.2 [5.9] | 1.1 [7] | -1.7 [5.1] | 3.2 [2.5] | 0.042 | - | - | 0.01 | 0.029 | < 0.01 | < 0.01 |
|  | PAFc | -2.7 [3.2] | -3 [2.5] | -0.6 [4] | -3.2 [2.3] | < 0.01 | - | < 0.01 | < 0.01 | - | - | < 0.01 |
|  | ROM | 5.4 [4] | 5.7 [1.8] | 4.1 [3.3] | 6.3 [2.5] | - | - | - | - | - | - | < 0.01 |
| Transverse | PATa | 2.5 [5.8] | 2 [4.3] | 0.2 [4.6] | 2.9 [4.2] | - | - | - | - | - | - | 0.045 |
|  | PATb | 3.8 [4.9] | 3.2 [3.5] | 4.2 [7.2] | 3.1 [3.2] | - | - | - | - | - | - | - |
|  | PATc | -3.3 [3.9] | -4.5 [5.7] | -2.3 [5] | -3.1 [3.5] | - | - | - | - | - | - | - |
|  | ROM | 8.1 [4] | 7.3 [4.2] | 6.6 [5.2] | 7.2 [3.5] | - | - | - | - | 0.011 | - | - |
| Hip | | | | | | | | | | | | |
| Sagittal | HASa | 35.1 [6.9] | 23.6 [8.1] | 26.5 [5.2] | 29.1 [8.3] | < 0.01 | < 0.01 | < 0.01 | < 0.01 | < 0.01 | < 0.01 | - |
|  | HASb | -4.4 [8.6] | -15.5 [7.3] | -10.2 [10.6] | -13.4 [9.2] | < 0.01 | < 0.01 | < 0.01 | < 0.01 | < 0.01 | - | < 0.01 |
|  | HASc | 37.2 [6.8] | 24.8 [7.1] | 27.8 [7.4] | 29.5 [7.9] | < 0.01 | < 0.01 | < 0.01 | < 0.01 | < 0.01 | < 0.01 | - |
|  | ROM | 42 [7.1] | 40.6 [7.9] | 36.2 [8.4] | 43.6 [6.5] | < 0.01 | - | < 0.01 | < 0.01 | - | 0.026 | < 0.01 |
| Frontal | HAFa | 0.9 [6.5] | 3.3 [7.7] | 2.9 [6] | 1.7 [4.2] | - | - | - | - | - | - | - |
|  | HAFb | 5.8 [5.9] | 8.8 [7.8] | 5.3 [5.7] | 7.3 [3.2] | - | - | - | - | - | - | 0.044 |
|  | HAFc | -2.8 [6.6] | -4.4 [6.1] | -0.8 [6.9] | -3.1 [3.8] | - | - | - | - | - | - | < 0.01 |
|  | ROM | 8.9 [4.9] | 11.1 [5.6] | 7.3 [3.8] | 10.6 [3.3] | 0.011 | - | 0.006 | 0.03 | < 0.01 | - | < 0.01 |
| Knee | | | | | | | | | | | | |
| Sagittal | KASa | 9 [7.7] | 1.7 [7.9] | 5.5 [6.2] | 6.1 [5.6] | < 0.01 | < 0.01 | - | - | 0.019 | 0.014 | - |
|  | KASb | 13.6 [8.1] | 8 [10.8] | 7.5 [12.9] | 14.9 [6.7] | < 0.01 | < 0.01 | - | < 0.01 | - | < 0.01 | < 0.01 |
|  | KASc | 5.7 [8.2] | 1.3 [6.2] | 0.7 [13.4] | -0.1 [6.1] | < 0.01 | < 0.01 | - | < 0.01 | < 0.01 | - | - |
|  | KASd | 48.8 [9.4] | 49 [6] | 41.2 [12.3] | 54.5 [8] | < 0.01 | - | < 0.01 | < 0.01 | < 0.01 | < 0.01 | < 0.01 |
|  | ROM | 43.6 [10.2] | 49.4 [7.5] | 43.6 [14.4] | 55.6 [4.9] | < 0.01 | < 0.01 | < 0.01 | - | < 0.01 | < 0.01 | < 0.01 |
| Frontal | KAFa | 1.1 [7.2] | -1.5 [16.6] | -0.9 [8.2] | -2 [3.8] | - | - | - | - | < 0.01 | - | - |
|  | KAFb | 4.2 [7.3] | 2.1 [17.9] | 1.4 [9.3] | 0.8 [4] | - | - | - | - | < 0.01 | - | - |
|  | KAFc | 1.4 [8.5] | 0 [18.3] | 0.9 [9.2] | -1.6 [4.2] | - | - | - | - | < 0.01 | - | - |
|  | ROM | 8.9 [6.9] | 7.2 [3.6] | 5.1 [4.8] | 5.1 [3.8] | < 0.01 | - | - | < 0.01 | < 0.01 | 0.038 | - |
| Ankle | | | | | | | | | | | | |
| Sagittal | AASa | 0.6 [3.5] | 1.1 [3.7] | 1.5 [4.6] | 0.4 [4.6] | - | - | - | - | - | - | - |
|  | AASb | -2.3 [3.8] | -4.4 [4.9] | 0.1 [7.1] | -2.4 [3.7] | 0.01 | 0.029 | 0.007 | - | - | - | < 0.01 |
|  | AASc | 15.8 [2.9] | 14.3 [5.5] | 13.9 [5.9] | 8.6 [3.4] | - | - | - | - | < 0.01 | < 0.01 | < 0.01 |
|  | AASd | -9.1 [8.4] | -10 [8.2] | -2.8 [5] | -12.5 [8.5] | < 0.01 | - | < 0.01 | < 0.01 | < 0.01 | - | < 0.01 |
|  | ROM | 25.2 [6.1] | 25.7 [6.5] | 20.5 [6.3] | 27.6 [5.4] | < 0.01 | - | < 0.01 | < 0.01 | - | - | < 0.01 |
| Foot | | | | | | | | | | | | |
| Progression | FPAa | -11 [7.8] | -10.6 [4.7] | -15.3 [5.5] | -11.1 [5.7] | < 0.01 | - | < 0.01 | < 0.01 | - | - | < 0.01 |
|  | FPAb | -7.1 [7.5] | -8 [5.5] | -12.4 [5] | -8 [5.7] | < 0.01 | - | < 0.01 | < 0.01 | - | - | < 0.01 |
|  | FPAc | -10 [8.7] | -11 [5] | -15.2 [4.4] | -11.1 [6.1] | < 0.01 | - | < 0.01 | < 0.01 | - | - | < 0.01 |
|  | FPAd | -4.2 [10] | -5.9 [4] | -9.9 [6.4] | -4.5 [6.6] | < 0.01 | - | < 0.01 | < 0.01 | - | - | < 0.01 |
|  | ROM | 12 [5.1] | 11.9 [5.1] | 9.7 [3.5] | 13.2 [5.9] | - | - | - | - | - | - | < 0.01 |

*KW stands for Kruskall-Walis tests between clusters (performed for continuous features) and CHI2 stands for the Chi-square test performed between clusters for proportion features. Between clusters comp. regroups the post-hoc tests (Wilcoxon or Chi2). Comp. with Control Group shows the comparison between cluster and CG (Wilcoxon or Chi2).*
