# Supplementary material for: Comprehensive analysis of total knee arthroplasty kinematics and functional recovery: Exploring full-body gait deviations in patients with knee osteoarthritis
Source: PLoS One. 2024 Dec 5;19(12):e0314991. doi: 10.1371/journal.pone.0314991 (PMC11620450; doi:10.1371/journal.pone.0314991)
Supplement: S3 Table — (DOCX) [file pone.0314991.s003.docx]

**Supporting Information 3.1: Spatio-temporal Parameters (median [IQR]) before surgery for patients, Clusters and Control Group.**

| **Features** | **All Patients (n=100)** | **Cluster 1**  **(n=59)** | **Cluster 2**  **(n=20)** | **Cluster 3**  **(n=21)** | **Control Group** | **KW or**  **CHI2** | **Between Clusters Comp.** | | | **Comp. with Control Group** | | |
| --- | --- | --- | --- | --- | --- | --- | --- | --- | --- | --- | --- | --- |
|  |  |  |  |  |  |  | **1 vs 2** | **2 vs 3** | **3 vs 1** | **CL1** | **CL2** | **CL3** |
| Walking Speed (m/s) | 1.0 [0.3] | 1.0 [0.2] | 1.0 [0.2] | 0.7 [0.2] | 1.1 [0.2] | < 0.01 | - | < 0.01 | < 0.01 | < 0.01 | 0.037 | < 0.01 |
| Stride Time (s) | 1.1 [0.2] | 1.1 [0.1] | 1.1 [0.1] | 1.3 [0.1] | 1.1 [0.1] | < 0.01 | - | < 0.01 | < 0.01 | - | - | < 0.01 |
| Stride Length (m) | 1.1 [0.2] | 1.2 [0.2] | 1.1 [0.1] | 0.9 [0.3] | 1.3 [0.2] | < 0.01 | - | < 0.01 | < 0.01 | < 0.01 | < 0.01 | < 0.01 |
| Cadence (step/min) | 105.3 [16.7] | 106.4 [11.8] | 111 [9.8] | 89.7 [9.2] | 109.2 [9.9] | < 0.01 | - | < 0.01 | < 0.01 | - | - | < 0.01 |
| Step Time (s) | 0.6 [0.1] | 0.6 [0.1] | 0.5 [0] | 0.7 [0.1] | 0.6 [0.1] | < 0.01 | < 0.01 | < 0.01 | < 0.01 | - | - | < 0.01 |
| Step Length (m) | 0.6 [0.1] | 0.6 [0.1] | 0.6 [0.1] | 0.5 [0.1] | 0.6 [0.1] | < 0.01 | - | < 0.01 | < 0.01 | < 0.01 | < 0.01 | < 0.01 |
| Step Width (m) | 0.1 [0.1] | 0.1 [0.1] | 0.1 [0] | 0.1 [0.1] | 0.1 [0] | - | - | - | - | < 0.01 | - | < 0.01 |
| Foot Off (%) | 62.5 [3.8] | 62.3 [2.4] | 62.3 [3.1] | 65.8 [6.6] | 61.1 [2.1] | 0.012 | - | 0.015 | 0.005 | < 0.01 | 0.012 | < 0.01 |
| Single Support (%) | 36.2 [3.7] | 36.5 [2.9] | 37.3 [2.6] | 31.4 [3.4] | 38.8 [2.2] | < 0.01 | - | < 0.01 | < 0.01 | < 0.01 | < 0.01 | < 0.01 |
| Double Support (%) | 26.5 [6.3] | 25.9 [4.4] | 24.1 [5.7] | 34.5 [9.8] | 22.4 [3.9] | < 0.01 | - | < 0.01 | < 0.01 | < 0.01 | < 0.01 | < 0.01 |

*KW* stands for Kruskall-Walis tests between clusters (performed for continuous features) and CHI2 stands for the Chi-square test performed between clusters for proportion features. Between clusters comparison regroups the post-hoc tests (Wilcoxon or Chi2). Comp. with Control Group shows the comparison between cluster and CG (Wilcoxon or Chi2).

**Supporting Information 3.2: Spatio-temporal Parameters (median [IQR]) one year after surgery for patients, Clusters and Control Group.**

| **Features** | **All Patients(n=100)** | **Cluster 1**  **(n=59)** | **Cluster 2**  **(n=20)** | **Cluster 3**  **(n=21)** | **Control Group** | **KW or**  **CHI2** | **Between Clusters Comp.** | | | **Comp. with Control Group** | | |
| --- | --- | --- | --- | --- | --- | --- | --- | --- | --- | --- | --- | --- |
|  |  |  |  |  |  |  | **1 vs 2** | **2 vs 3** | **3 vs 1** | **CL1** | **CL2** | **CL3** |
| Walking Speed (m/s) | 1.1 [0.3] | 1.1 [0.2] | 1.1 [0.2] | 0.8 [0.3] | 1.1 [0.2] | < 0.01 | - | < 0.01 | < 0.01 | - | - | < 0.01 |
| Stride Time (s) | 1.1 [0.1] | 1.1 [0.1] | 1 [0.1] | 1.2 [0.1] | 1.1 [0.1] | < 0.01 | < 0.01 | < 0.01 | < 0.01 | - | < 0.01 | < 0.01 |
| Stride Length (m) | 1.2 [0.2] | 1.2 [0.2] | 1.2 [0.1] | 1 [0.2] | 1.3 [0.2] | < 0.01 | - | < 0.01 | < 0.01 | 0.022 | 0.024 | < 0.01 |
| Cadence (step/min) | 108.4 [12] | 108.9 [10.1] | 114.9 [6.5] | 99.7 [11.5] | 109.2 [9.9] | < 0.01 | < 0.01 | < 0.01 | < 0.01 | - | < 0.01 | < 0.01 |
| Step Time (s) | 0.6 [0.1] | 0.5 [0] | 0.5 [0] | 0.6 [0.1] | 0.6 [0.1] | < 0.01 | - | < 0.01 | < 0.01 | - | < 0.01 | < 0.01 |
| Step Length (m) | 0.6 [0.1] | 0.6 [0.1] | 0.6 [0] | 0.5 [0.1] | 0.6 [0.1] | < 0.01 | - | < 0.01 | < 0.01 | - | 0.048 | < 0.01 |
| Step Width (m) | 0.1 [0] | 0.1 [0.1] | 0.1 [0] | 0.1 [0.1] | 0.1 [0] | - | - | - | - | < 0.01 | - | < 0.01 |
| Foot Off (%) | 62.5 [3.6] | 62.3 [3] | 62.2 [3.3] | 64.9 [5.3] | 61.1 [2.1] | < 0.01 | - | < 0.01 | < 0.01 | < 0.01 | - | < 0.01 |
| Single Support (%) | 37.2 [2.8] | 37.4 [2.3] | 37.8 [2.3] | 35.2 [4.2] | 38.8 [2.2] | < 0.01 | - | < 0.01 | < 0.01 | < 0.01 | 0.011 | < 0.01 |
| Double Support (%) | 25.3 [5.7] | 24.9 [4.7] | 24.5 [6.4] | 28.5 [9.1] | 22.4 [3.9] | < 0.01 | - | < 0.01 | < 0.01 | < 0.01 | - | < 0.01 |

*KW stands for Kruskall-Walis tests between clusters (performed for continuous features) and CHI2 stands for the Chi-square test performed between clusters for proportion features. Between clusters comparison regroups the post-hoc tests (Wilcoxon or Chi2). Comp. with Control Group shows the comparison between cluster and CG (Wilcoxon or Chi2).*
