# Supplementary material for: Comprehensive analysis of total knee arthroplasty kinematics and functional recovery: Exploring full-body gait deviations in patients with knee osteoarthritis
Source: PLoS One. 2024 Dec 5;19(12):e0314991. doi: 10.1371/journal.pone.0314991 (PMC11620450; doi:10.1371/journal.pone.0314991)
Supplement: S4 Table — (DOCX) [file pone.0314991.s004.docx]

**Supporting Information 4:** Kinematic Features (median [IQR]) before and one-year post surgery for Clusters and Control Group. *Pre vs. one year post-surgery* regroups the pre-post comparison within clusters (Wilcoxon). *One year post surgery vs. control proup* shows the comparison between clusters one year after surgery and CG (Wilcoxon).

|  | **Feat.**  **(deg)** | **Cluster 1 (n=59)** | | **Cluster 2 (n=20)** | | **Cluster 3 (n=21)** | | **Control Group** | **Pre vs. one year post surgery** | | | **One year post surgery vs. control proup** | | |
| --- | --- | --- | --- | --- | --- | --- | --- | --- | --- | --- | --- | --- | --- | --- |
|  |  | **Pre surgery** | **One year post surgery** | **Pre surgery** | **One year post surgery** | **Pre surgery** | **One year post surgery** |  |  |  |  |  |  |  |
|  |  |  |  |  |  |  |  |  | **CL1** | **CL2** | **CL3** | **CL1** | **CL2** | **CL3** |
| **Thorax** | | | | | | | | | | | | | | |
| Sagittal | TASa | 3.4 [7] | 3.1 [6.3] | 7.6 [8.4] | 7.9 [8.4] | 6 [8] | 4.5 [8.1] | 2.4 [6.9] | - | - | - | - | < 0.01 | 0.029 |
|  | TASb | 2.7 [7.2] | 1.7 [6.3] | 5.8 [8] | 6.2 [7.8] | 5.1 [7.7] | 3.5 [8.3] | 1.5 [6.9] | - | - | - | - | 0.014 | 0.04 |
|  | TASc | 4.6 [6.8] | 2.8 [6.7] | 8.4 [8.2] | 8.3 [7.2] | 7.2 [9.4] | 5.8 [8.1] | 3.2 [7.4] | - | - | - | - | 0.02 | 0.018 |
|  | TASd | 2.4 [7.7] | 1.6 [6.4] | 5.5 [8.1] | 6.1 [6.8] | 4 [7.8] | 3.6 [6.9] | 1.3 [7.2] | - | - | - | - | 0.021 | 0.04 |
|  | TASe | 2.7 [1.4] | 2.4 [1.1] | 2.5 [1.6] | 2.6 [1.2] | 4 [2.2] | 2.5 [1.6] | 2.4 [0.9] | 0.027 | - | 0.035 | - | - | - |
| Frontal | TAFa | -0.2 [2.8] | 0.3 [3] | -0.6 [2.5] | -0.2 [2.4] | 0.3 [2.4] | -1.1 [3] | -0.3 [2.6] | - | - | - | - | - | - |
|  | TAFb | -2.6 [3.8] | -1.9 [2.8] | -2 [4] | -1.9 [2.2] | -3.9 [3.6] | -2.7 [3.6] | -1.5 [3] | 0.049 | - | - | - | - | 0.021 |
|  | TAFc | 1.3 [3.8] | 1.6 [3.4] | 1.6 [1.9] | 1.7 [2.4] | 1.9 [2.4] | 1.2 [2.9] | 1.6 [2.8] | - | - | - | - | - | - |
|  | TAFd | 4.2 [2.7] | 3.7 [2.7] | 3.5 [2.7] | 3.6 [2.8] | 5.2 [4.6] | 3.9 [3.8] | 2.6 [2.2] | - | - | - | - | - | - |
| Transverse | TATa | -2.1 [4.2] | -1.8 [3.4] | -3 [3.4] | -2.7 [4.6] | -2.1 [3.1] | -2 [4.7] | -1.9 [4.1] | - | - | - | - | - | - |
|  | TATb | 0.8 [4] | 1.5 [4.4] | 1.6 [3.3] | 1.2 [2.6] | 1 [4.6] | 0.7 [4.5] | 2.1 [4.6] | - | - | - | - | - | - |
|  | TATc | 5.4 [3.6] | 5.8 [3] | 5.4 [3.2] | 5.2 [3.2] | 6.3 [2.4] | 6.7 [2.5] | 5.5 [4.3] | - | - | - | - | - | - |
| Pelvis | | | | | | | | | | | | | | |
| Sagittal | PASa | 14.1 [5.4] | 11.1 [7.8] | 4.5 [4.7] | 5.3 [8.1] | 8.5 [3.4] | 6.6 [9.5] | 6.7 [5.7] | < 0.01 | - | - | < 0.01 | - | - |
|  | PASb | 13.2 [4.9] | 9.9 [7.2] | 3.2 [3.7] | 4.4 [7.2] | 8.2 [4] | 5.2 [10.5] | 6.4 [6.1] | < 0.01 | - | - | < 0.01 | 0.049 | - |
|  | PASc | 15.2 [3.9] | 10.8 [6.8] | 5.9 [4.2] | 6.1 [9] | 11 [5.2] | 8.7 [9.6] | 7.9 [6] | < 0.01 | - | - | < 0.01 | - | - |
|  | PASd | 12.4 [4.6] | 9.4 [7.3] | 3.5 [4.1] | 4.1 [8.1] | 7.6 [4.1] | 5.7 [9.2] | 6 [6.2] | < 0.01 | - | - | < 0.01 | - | - |
|  | PASe | 2.9 [1.7] | 2.5 [1.1] | 2.8 [1.5] | 2.4 [1.3] | 3.5 [2.3] | 2.6 [1.4] | 2.2 [1.1] | - | - | - | < 0.01 | - | 0.012 |
| Frontal | PAFa | -0.2 [2.9] | -0.2 [2.2] | -0.5 [3.1] | 0.4 [2.8] | 0.2 [3.7] | -0.1 [1.9] | -0.1 [2.1] | - | - | - | - | - | - |
|  | PAFb | 2.2 [5.9] | 1.9 [5.7] | 1.1 [7] | 3 [6.9] | -1.7 [5.1] | 1 [6.2] | 3.2 [2.5] | - | - | 0.047 | 0.019 | - | - |
|  | PAFc | -2.7 [3.2] | -3 [2.6] | -3 [2.5] | -2.7 [2.2] | -0.6 [4] | -1.1 [3] | -3.2 [2.3] | - | - | - | - | - | < 0.01 |
|  | PAFd | 5.4 [4] | 5.6 [3.4] | 5.7 [1.8] | 7.1 [2] | 4.1 [3.3] | 3.9 [2.7] | 6.3 [2.5] | - | 0.026 | - | - | - | < 0.01 |
| Transverse | PATa | 2.5 [5.8] | 2.6 [4.1] | 2 [4.3] | 2.4 [2.4] | 0.2 [4.6] | 0.9 [5.1] | 2.9 [4.2] | - | - | - | - | - | - |
|  | PATb | 3.8 [4.9] | 3.3 [3.5] | 3.2 [3.5] | 2.6 [2.5] | 4.2 [7.2] | 3.4 [5.1] | 3.1 [3.2] | - | - | - | - | - | - |
|  | PATc | -3.3 [3.9] | -3.5 [4.5] | -4.5 [5.7] | -5 [5.4] | -2.3 [5] | -2.6 [3.9] | -3.1 [3.5] | - | - | - | - | - | - |
|  | PATd | 8.1 [4] | 9 [4.1] | 7.3 [4.2] | 7.9 [4.1] | 6.6 [5.2] | 6.9 [2.4] | 7.2 [3.5] | - | - | - | < 0.01 | - | - |
| Hip | | | | | | | | | | | | | | |
| Sagittal | HASa | 35.1 [6.9] | 33.2 [9.7] | 23.6 [8.1] | 23 [9.2] | 26.5 [5.2] | 25.5 [12.7] | 29.1 [8.3] | 0.033 | - | - | < 0.01 | 0.012 | - |
|  | HASb | -4.4 [8.6] | -9.8 [11] | -15.5 [7.3] | -17.6 [8.9] | -10.2 [10.6] | -9.3 [9.1] | -13.4 [9.2] | < 0.01 | - | - | < 0.01 | - | < 0.01 |
|  | HASc | 37.2 [6.8] | 35 [9.6] | 24.8 [7.1] | 24.9 [10.3] | 27.8 [7.4] | 27.7 [14] | 29.5 [7.9] | 0.034 | - | - | < 0.01 | 0.049 | - |
|  | HASd | 42 [7.1] | 43.5 [8.8] | 40.6 [7.9] | 42.4 [6.4] | 36.2 [8.4] | 39.5 [8.7] | 43.6 [6.5] | - | - | - | - | - | < 0.01 |
| Frontal | HAFa | 0.9 [6.5] | 3.9 [5.5] | 3.3 [7.7] | 5.1 [6.8] | 2.9 [6] | 3.1 [4.9] | 1.7 [4.2] | < 0.01 | - | - | < 0.01 | < 0.01 | 0.015 |
|  | HAFb | 5.8 [5.9] | 9.1 [5.4] | 8.8 [7.8] | 11.5 [6.5] | 5.3 [5.7] | 8.5 [2.8] | 7.3 [3.2] | < 0.01 | - | < 0.01 | < 0.01 | < 0.01 | < 0.01 |
|  | HAFc | -2.8 [6.6] | -1.3 [4] | -4.4 [6.1] | -2.6 [6.5] | -0.8 [6.9] | 1.8 [4.6] | -3.1 [3.8] | 0.01 | - | - | < 0.01 | - | < 0.01 |
|  | HAFd | 8.9 [4.9] | 9.8 [4.2] | 11.1 [5.6] | 11.9 [5.9] | 7.3 [3.8] | 8.6 [3.3] | 10.6 [3.3] | - | - | - | - | - | < 0.01 |
| Knee | | | | | | | | | | | | | | |
| Sagittal | KASa | 9 [7.7] | 5.9 [5] | 1.7 [7.9] | 3.2 [6.5] | 5.5 [6.2] | 6.8 [7.3] | 6.1 [5.6] | < 0.01 | - | - | - | 0.016 | - |
|  | KASb | 13.6 [8.1] | 13.5 [6.6] | 8 [10.8] | 9.5 [6.9] | 7.5 [12.9] | 9.6 [9.8] | 14.9 [6.7] | - | - | - | - | < 0.01 | < 0.01 |
|  | KASc | 5.7 [8.2] | 3.6 [6.3] | 1.3 [6.2] | 2.1 [6.5] | 0.7 [13.4] | 0.5 [7.9] | -0.1 [6.1] | - | - | - | < 0.01 | - | - |
|  | KASd | 48.8 [9.4] | 50.7 [8.5] | 49 [6] | 51 [8] | 41.2 [12.3] | 45 [10.9] | 54.5 [8] | - | - | - | < 0.01 | < 0.01 | < 0.01 |
|  | KASe | 43.6 [10.2] | 47.3 [9.8] | 49.4 [7.5] | 48.5 [9.7] | 43.6 [14.4] | 46.3 [10.4] | 55.6 [4.9] | 0.014 | - | - | < 0.01 | < 0.01 | < 0.01 |
| Frontal | KAFa | 1.1 [7.2] | -3.8 [5.4] | -1.5 [16.6] | -4.6 [7.5] | -0.9 [8.2] | -3.7 [5] | -2 [3.8] | < 0.01 | - | 0.044 | < 0.01 | - | 0.015 |
|  | KAFb | 4.2 [7.3] | -1.6 [5.8] | 2.1 [17.9] | -3 [7.2] | 1.4 [9.3] | -1.6 [6] | 0.8 [4] | < 0.01 | - | 0.047 | < 0.01 | - | < 0.01 |
|  | KAFc | 1.4 [8.5] | -4.3 [5.1] | 0 [18.3] | -4.1 [9.2] | 0.9 [9.2] | -3.7 [4.9] | -1.6 [4.2] | < 0.01 | - | 0.016 | < 0.01 | - | 0.011 |
|  | KAFe | 8.9 [6.9] | 5.9 [4.5] | 7.2 [3.6] | 5.6 [6] | 5.1 [4.8] | 5.3 [3.2] | 5.1 [3.8] | < 0.01 | - | - | - | - | - |
| Ankle | | | | | | | | | | | | | | |
| Sagittal | AASa | 0.6 [3.5] | -1.7 [4.4] | 1.1 [3.7] | -0.4 [2.3] | 1.5 [4.6] | 0.5 [3.3] | 0.4 [4.6] | 0.013 | - | - | - | - | - |
|  | AASb | -2.3 [3.8] | -4.2 [3.5] | -4.4 [4.9] | -4 [3.9] | 0.1 [7.1] | -2.2 [8.3] | -2.4 [3.7] | < 0.01 | - | - | < 0.01 | 0.021 | - |
|  | AASc | 15.8 [2.9] | 16 [3.9] | 14.3 [5.5] | 15 [3.8] | 13.9 [5.9] | 15 [4.6] | 8.6 [3.4] | - | - | - | < 0.01 | < 0.01 | < 0.01 |
|  | AASd | -9.1 [8.4] | -12 [9] | -10 [8.2] | -11 [5.9] | -2.8 [5] | -5.8 [10.5] | -12.5 [8.5] | - | - | - | - | - | < 0.01 |
|  | AASe | 25.2 [6.1] | 27 [7.6] | 25.7 [6.5] | 27.4 [5.2] | 20.5 [6.3] | 24.6 [7.4] | 27.6 [5.4] | - | - | < 0.01 | - | - | - |
| Foot | | | | | | | | | | | | | | |
| Progression | FPAa | -11 [7.8] | -10.6 [9.2] | -10.6 [4.7] | -10.7 [4.8] | -15.3 [5.5] | -13.4 [7.5] | -11.1 [5.7] | - | - | - | - | - | < 0.01 |
|  | FPAb | -7.1 [7.5] | -8.2 [8.9] | -8 [5.5] | -8 [6] | -12.4 [5] | -12.8 [8.2] | -8 [5.7] | - | - | - | - | - | < 0.01 |
|  | FPAc | -10 [8.7] | -10.9 [10.1] | -11 [5] | -11.2 [6.1] | -15.2 [4.4] | -14.9 [11] | -11.1 [6.1] | - | - | - | - | - | < 0.01 |
|  | FPAd | -4.2 [10] | -5.6 [11.1] | -5.9 [4] | -6.6 [5.4] | -9.9 [6.4] | -10.5 [7.3] | -4.5 [6.6] | 0.019 | - | - | - | 0.035 | < 0.01 |
|  | FPAe | 12 [5.1] | 12.5 [6.5] | 11.9 [5.1] | 10.8 [4.8] | 9.7 [3.5] | 11.8 [5.1] | 13.2 [5.9] | - | - | - | - | - | - |
