# Supplementary material for: Comprehensive analysis of total knee arthroplasty kinematics and functional recovery: Exploring full-body gait deviations in patients with knee osteoarthritis
Source: PLoS One. 2024 Dec 5;19(12):e0314991. doi: 10.1371/journal.pone.0314991 (PMC11620450; doi:10.1371/journal.pone.0314991)
Supplement: Attachment — Submitted filename: Clustering - AnswerToReviewers - Review_1.docx [file pone.0314991.s009.docx]

PONE-D-24-24263

Comprehensive Analysis of Total Knee Arthroplasty Kinematics and Functional Recovery: Exploring Full-Body Compensations in Patients with Knee Osteoarthritis

PLOS ONE

Dear Dr. Gasparutto,

Thank you for submitting your manuscript to PLOS ONE. After careful consideration, we feel that it has merit but does not fully meet PLOS ONE’s publication criteria as it currently stands. Therefore, we invite you to submit a revised version of the manuscript that addresses the points raised during the review process.

Some concerns were raised by the two reviewers. One concern is that the patients were analyzed at different times with different measurement systems and that there is a lack of information on the implants used and that both unilaterally and bilaterally affected patients were included in the study. These points should be addressed in more detail in the discussion and the limitations section.

These points are now considered in the paper and were answered in detail in the reviewers’ comment section. As a quick overview:

- Recent studies showed no difference in terms of accuracy between modern measurement systems,
- We added information on implants for all patients. Although there were differences in implant type between the two datasets, the proportion of patients belonging to one dataset or the other was the same between each clusters and for the cohort. Additionaly, there were no differences in terms of type of implant between clusters. The analysis was blind to the type of surgery or implant.
- We chose to mix unilateral and bilateral patients to match with the patient’s population planned for TKA.

Another issue that was raised was that the authors identify three patient groups according to their strategy to walk but have not clarifies how they know if these strategies are compensatory mechanisms. Could it be that these people just walked in a particular manner but that it is not a compensatory mechanism? While comparisons are made to the control group it is unclear whether clusters also exist in healthy patients and whether any of those clusters is similar to the TKA clusters.

This question seems to be on point, and we likely have misused this term. Indeed, since their identification would require additional clinical data that we don’t have at our disposal in this database, it was chosen to only consider “gait deviations” in the paper. In this way, the objective stated fits better the design of the study. Thus, the term “compensation” was replaced by “gait deviation” in the title and throughout the manuscript.

A final major issue is that the authors hypothesize that three groups can be considered: high functional, low functional, limping group, but do not provide any criteria to be used to assess function. Without these criteria, it is unclear that the hypothesis can be tested.

This hypothesis was based on a previous study by Young-Shand that mentioned high and low function groups in a population of patients undergoing TKA. The criteria used was simply the amount of deviation with respect to the control group, especially in terms of gait speed, a key indicator of functional levels [1-3]. However, we did not set thresholds for high or low functional levels. To phrase this appreciation in a relative manner, we are now talking about a “higher” and a “lower” functional group throughout the manuscript.

This was specified in the introduction:
L97: “We hypothesize that multiple clusters will emerge with a higher functional group, a lower functional group, and a limping group with upper body gait deviations. The level of function will be assessed by comparison to with the control group in terms of walking speed, gait kinematics, and spatio-temporal parameters.”

Also, no statistical analyses are presented to confirm that the three obtained groups have different kinematics and spatio-temporal parameters of gait.

We disagree with this point. We performed multiple statistical analyses described between L185 and L193 in the “Data Analysis” section in the results, in table 1 and under visual representation in figures 1, 2 and 3. All results were not reported in the text to make the paper easier to read and understand. However, all tests were reported in the supporting information 1, 2, 3 and 4. We did notice a typo in supporting information 4 so we updated a new one with this revision.

In this study, the clusters of patients were compared between each other before surgery, to the control group before and after surgery and finally their pre-surgery visit was compared to their post-surgery one to assess their evolution. The outcomes compared were the clinical data (sex, age, height, BMI, % bilateral, WOMAC, SF12), the kinematic features of the thorax, pelvis, hip, knee, ankle, and foot progression as well as the spatio-temporal parameters including walking speed, stride time, stride length, cadence, step time, step length, step width, time of foot off, percentage of single support and percentage of double support.

As explained in the section Data Analysis:
L185: “After data clustering, the clusters pre-surgery were compared to each other in terms of clinical data, kinematics features, and spatio-temporal data. The analysis was blind to the type of surgery or implant. The Kruskall-Wallis test with post-hoc Wilcoxon tests was used to compare the multiple clusters for all continuous data. The Chi2 test was used for proportions (satisfied or not, side of surgery, bilateral knee OA, sex). The evolution of each cluster was assessed by comparing the clinical data, kinematics features, and spatio-temporal data measured pre-surgery to the ones measured one year after surgery with Wilcoxon tests. Finally, each cluster was compared before and after surgery to the control group with a Wilcoxon test. Statistical significance was set at p < 0.05.”

Journal requirements:

 1. When submitting your revision, we need you to address these additional requirements.

 Please ensure that your manuscript meets PLOS ONE's style requirements, including those for file naming. The PLOS ONE style templates can be found at

<https://journals.plos.org/plosone/s/file?id=wjVg/PLOSOne_formatting_sample_main_body.pdf> and

<https://journals.plos.org/plosone/s/file?id=ba62/PLOSOne_formatting_sample_title_authors_affiliations.pdf>.

 2. Thank you for stating the following financial disclosure:

 [This study was funded by the "Fondation pour la recherche ostéoarticulaire" of Geneva (Switzerland).].

Please state what role the funders took in the study.  If the funders had no role, please state: ""The funders had no role in study design, data collection and analysis, decision to publish, or preparation of the manuscript.""

If this statement is not correct you must amend it as needed.

Please include this amended Role of Funder statement in your cover letter; we will change the online submission form on your behalf.

The full statement was included in the cover letter submitted with the review.

  3. Thank you for stating the following in the Acknowledgments Section of your manuscript:

[This work was supported by the Department of orthopaedic surgery and trauma care at the Geneva University Hospitals and by the “Fondation pour la recherche ostéoarticulaire” of Geneva. The funding source did not play a role in the design of this study.]

We note that you have provided funding information that is not currently declared in your Funding Statement. However, funding information should not appear in the Acknowledgments section or other areas of your manuscript. We will only publish funding information present in the Funding Statement section of the online submission form.

Please remove any funding-related text from the manuscript and let us know how you would like to update your Funding Statement. Currently, your Funding Statement reads as follows:

 [This study was funded by the "Fondation pour la recherche ostéoarticulaire" of Geneva (Switzerland).]

This section was removed from the manuscript.

 Please include your amended statements within your cover letter; we will change the online submission form on your behalf.

 4. Please provide a complete Data Availability Statement in the submission form, ensuring you include all necessary access information or a reason for why you are unable to make your data freely accessible. If your research concerns only data provided within your submission, please write "All data are in the manuscript and/or supporting information files" as your Data Availability Statement.

To avoid sharing unpublished data, and, following the questions in the submission (‘Additional data availability information - Tick here if the URLs/accession numbers/DOIs will be available only after acceptance of the manuscript for publication’) we chose to fully open the data at the end of the submission.

The repository is shared on the platform Yareta according to the FAIR principles at the following DOI: <https://doi.org/10.26037/yareta:4xnhbsiyqrbihlep2nst2u2hyq>

We set the repository to fully public since this seems necessary for the review process.

5. Review Comments to the Author

Please use the space provided to explain your answers to the questions above. You may also include additional comments for the author, including concerns about dual publication, research ethics, or publication ethics. (Please upload your review as an attachment if it exceeds 20,000 characters)

**Reviewer #1:** This is a solid study. An unsupervised approach has been chosen, to determine several clusters from full-body gait kinematic and spatio-temporal parameters in patients with unilateral and bilateral knee osteoarthritis (OA). Three functional groups of patients undergoing total knee arthroplasty (TKA) were identified. One low-functioning cluster regrouped mostly elderly women with the lowest function levels before and after surgery but with the most functional improvements after surgery. Two high-functioning clusters separated mainly by pelvis tilt (anteversion vs. retroversion), knee alignment (varus vs. neutral), and knee flexion during stance phase (flexum vs. extended) presented slight to no improvement after one year. The authors concluded that high functioning patients may benefit from TKA, mostly due to pain reduction, but may not see significant improvement of their function. On the contrary, patients with important functional limitation are more likely to improve both pain and functional outcomes.

Although it is an interesting study approach, I have some concerns about the generalizability of the study results. My main concern is that the patients were analyzed at different times with different measurement systems.

The type of camera of the VICON system (MX 3+) was missing and is now added (L148). These cameras have 0.3MP which is indeed low with respect to the Qualisys Oqus7+ with 12MP, however, both measurements were done with 12 cameras.

Topley et al. [4] performed an in-depth comparison of multiple modern optoelectronic systems and concluded that: “given the consistency in accuracy reported by all the systems included in the current assessment, it’s reasonable to assume that the current systems would eliminate errors associated with system hardware/software as an obstacle to sharing data between laboratories”.

The minimal resolution in Topley’s study was 1.3MP, thus it could be argued that the MX3+ have lower accuracy. However, Richards et al. [5] performed accuracy tests in 1999, on a former version of the VICON system (VICON 370) with only 6 cameras and found errors on a fixed 50mm distance of 0.062mm [0.183mm] (RMS [max]), of 0.129mm [0.557mm] on a 9cm distance between two markers on a moving plate and an error in angles of 1.4° [4.6°].

Moreover, Gorton et al [6] explored in 2009 the source of variability of human motion capture measurements by comparing 12 laboratories. The average instrumental error on a known distance was 1.2mm across labs with an average between lab deviation of only 0.9mm. When comparing instrumental errors to marker placement differences, they concluded that “marker placement differences between examiners are shown to be the most likely source of between site variability”.

Thus, since marker placement variability is way higher than between system variability, we believe that the difference between systems is negligible in our case since the measurements were performed at the same lab with the same markerset and same palpation method between datasets.

A specific section was added to the limitation section of the discussion:

L371: “Secondly, the cohort combined datasets assessed with different motion capture systems and generations. A recent study from 2020 by Topley et al. [4] showed that modern systems were equivalent in terms of instrumental errors and Gorton et al. [6] showed that the instrumental error was way lower than marker misplacement errors when assessing variability. Thus, since both databases were measured in the same laboratory, with the same number of cameras, with the same marker set and the same palpation technique for marker placement, we assume that the instrumental errors between systems did not impact the results of the study.”

Secondly, there is a lack of information on the implants used and both unilaterally and bilaterally affected patients were included. These points should be addressed in more detail in the discussion and the limitations section.

Additional details on the surgery were added in the manuscript:
L121 : “The surgical procedure and postoperative management have been previously described [7]. The surgery were done routinely by a medial parapatellar approach, with use of fully cemented implants. For the first dataset, most of the patients had a tibia first technique, using a mechanical alignment. A fixed bearing was used in all patients but in the case of ultra-congruent (UC) constraint. In the second dataset, a kinematic alignment was preferred when using TKA with a medial pivot polyethylene, with a femur first, caliper-measurement technique. Cementing technique has been always realised in a two-step manner, by means of antibiotic loaded PMMA (Palacos). Patella resurfacing has been carried out routinely for the second dataset, independently from the constraint used, given a tendency towards too many revision for anterior knee pain.”

The analysis was performed blindly to the type of implant or surgery. This was added to the manuscript.

L186: “The analysis was blind to the type of surgery or implant.”

Regarding the mix of unilateral and bilateral knee OA patients, studies are shared on the topic. Messier et al. [8] reported no differences in lower extremity kinematics or symmetry parameters while Creaby et al. [9] reported no differences in lower extremity kinematics by differences in terms of symmetry between unilateral and bilateral knee OA patients.

Given these studies, we chose to include both uni- and bi-lateral patients to be more representative of the population undergoing TKA. Indeed, in a study on prevalence of generalized osteoarthritis in knee OA patients, Forestier et al. [10] found 66% of patients with bilateral knee OA and 52% of patients with at least one out of the three definitions of generalised OA in the studied population of 305 patients. This point was added to the introduction, method, and discussion to clarify this question.

Introduction, L97: “We hypothesize that multiple clusters will emerge with a higher functional group, a lower functional group including mostly patients with bilateral knee OA, a limping group with upper body gait deviations.”

Method, L104: “One hundred patients planned for unilateral primary TKA due to end-stage knee OA were included in this study, along with 33 healthy participants recruited as a control group. Thirty-nine patients had bilateral knee OA.”

Discussion, L345: “Contrary to our hypothesis, there were no differences in terms of unilateral or bilateral knee affection between the three clusters. These results support studies by Creaby et al. [9] and Messier et al. [8] that agreed on the absence of differences in terms of lower limb kinematics between those two groups of patients, although their results differed on the symmetry parameters.”

The following are specific minor comments or recommendations:
.
ABSTRACT
• The authors stated that the knee was excluded from classification to focus on full-body kinematics (page 2, line 35). However, the results indicated two high-functioning clusters differentiated by pelvis tilt, sagittal knee alignment (varus vs. neutral), and knee flexion during stance phase (flexum vs. extended) (page 2, lines 38-42). Is this not a contradiction? Please also clarify/modify this point throughout the manuscript.

This is indeed what we did, the classification was not performed on the knee but on the kinematic features of the thorax, pelvis, hip, ankle and foot progression, as well as a selection of spatio-temporal parameters. The fact that differences in knee kinematics appear between those two groups does not seem to be a contradiction to us. Knee OA affects the knee biomechanics which in turn affects the full body. From our point of view, this is a key result of this study that suggests that different knee affection, here quantified with the kinematics, will affect differently the full body.

This point was underlined in the discussion:

L289: “Although the knee kinematics was excluded in the clustering process, CL1 and CL2 still presented differences in this outcome. This result reflects that, despite similar functional levels reflected by their similar walking speed, different knee phenotypes (varus/neutral) may have an impact on the full-body mechanics of the patients.”

1 INTRODUCTION
• Page 5, lines 77-78: I suggest being more precise: “...the external knee adduction moment”…

Thank you for this remark, this was added to the manuscript.

• Page 5, lines 80-82: I suggest adding a few references and being more precise how knee OA impacts patients gait.

This sentence has been completed with a reference and a short indication on the effect of knee OA on gait:

L80: “Indeed, patients with knee OA have been showed to present reduced gait speed and knee flexion range of motion [11] but it is not known how knee OA impacts the overall movement of the body, what adaptations patients employ to mitigate pain and maintain function, and whether such adaptations evolve or disappear with surgery.

• Page 5 and 6, lines 96-98: I suggest an uniform definition of the objectives/hypotheses, both in the abstract and here at the end of the Introduction.

The objectives at the end of the introduction were written in the same way as the abstract to clarify.

L93 : “The objective of this study were 1) assessing the impact of knee OA on full-body gait mechanics, 2) assessing whether potential gait deviations persist one year after TKA surgery, and 3) their potential impact on satisfaction.”

2 MATERIALS AND METHODS
Participant’s selection and characterstics
• How did the study group ensured that the reference group and the patient group (contralateral side for the unilateral affected patients) did not have asymptomatic knee osteoarthritis? X-rays were probably not possible for ethical reasons, were they?

There were no X-rays performed to avoid exposing controls to unnecessary radiations. We checked the inclusion-exclusion criteria mentioned in the manuscript (L106 to L112) during an interview before the measurement. Some participants of the control group may have had asymptomatic knee OA, to clarify, we added “symptomatic” to knee OA:

L109: “The healthy participants were matched in age and gender with the patient population and were included in the absence of symptomatic knee OA or other orthopaedic or neurologic problems potentially affecting gait or balance”.

• Page 6, line 113: …”and were performed at the same gait lab.” This information is redundant (see the first sentence of this paragraph).

This part of the sentence was removed.

• Page 6, lines 111-112: Please be more precise with the information regarding the follow-up measurement (e.g., mean and standard deviation / range).

Patients were measured with a postsurgery follow-up of 12.8+-0.6months [10.4 to 16.3 months]. This was added to the manuscript

L114: “The first dataset included 79 the second included 21 patients. Patients in both datasets underwent primary TKA and had a a follow-up of measurement at 1 year (12.8 ± 0.6months, range between 10.4 and 16.3months).”

• Page 6, line 117: Even though the authors list a reference here, it would be desirable for the reader to learn a little more about the implant used. So, were the same implants always used? Especially as the two measurement dates of the patients are very far apart (2010 – 2012 vs. 2018 – 2021). What effect could the type of implant have on the results?

This is a good point raised by the reviewer. After 2012 many new implants came into the market and we started using a medial pivot constraint design, while abandoning the UC constraint. On the other hand, we kept on using the PFC PS until recently, since no more available in our country, and replaced by the PERSONA knee design, in its PS version.

We provided with a table (Table 2) showing differences among the two datasets. We acknowledge that for the sake of research a more homogenous cohort would have been best fitting.

However, the percentage of patients from each dataset was the same in each clusters as in the cohort and, there was no differences in terms of implant types between the clusters. This suggests that the post-surgery differences between clusters may be due to the pre-surgery functional profiles. The fact that the clustering was performed on pre-surgery data may have helped tempering the implants’ influence.

We added the results of these tests to the main body of the paper:

Data Analysis – L188: “The Chi2 test was used for proportions (satisfied or not, side of surgery, bilateral knee OA, sex, type of implant, dataset)”

Results – L205: “There was no significant difference in proportion of patients from each dataset in each cluster, respectively 15.3%, 30% and 29% of patients from cohort 2 for CL1, CL2 and CL3 and with the porporition for the whole cohort (21%). There were differences in terms of implant type between datasets but there was no differences between clusters (Table 2).”

Discussion – L378: “Third, the cohorts presented differences in terms of implant which was mostly due to the evolution of the implant market. However, the percentage of patients from each dataset was the same in each clusters as in the cohort and, there was no differences in terms of implant types between the clusters. This suggests that the post-surgery differences between clusters may be due to the pre-surgery functional profiles. The fact that the clustering was performed on pre-surgery data may have helped tempering the implants’ influence.”

Gait measurements

• The Conventional Gait Model 1.0 was implemented in 2019. Please clarify which gait model (marker placement) was used for the first cohort (2010 – 2012) and discuss potential differences regarding the gait model.

The Conventional Gait Model can be traced back to 1972 [12]. This model has been widely used in clinics since the 70s-80s with various names (e.g. Plug-in-Gait model, Helen Hayes model, Newington model). The Conventional Gait Model name was introduced by Baker et al. [12] to clarify the definition and set a clear framework for the markerset and biomechanical models. Thus, this model was already used in 2010.

Please also discuss potential differences by using two motion capture systems (VICON vs. Qualisys).

The type of camera of the VICON system (MX 3+) was missing and is now added (L148). These cameras have 0.3MP which is indeed low with respect to the Qualisys Oqus7+ with 12MP, however, both measurements were done with 12 cameras.

Topley et al. [4] performed an in-depth comparison of multiple modern optoelectronic systems and concluded that: “given the consistency in accuracy reported by all the systems included in the current assessment, it’s reasonable to assume that the current systems would eliminate errors associated with system hardware/software as an obstacle to sharing data between laboratories”.

The minimal resolution in Topley’s study was 1.3MP, thus it could be argued that the MX3+ have lower accuracy. However, Richards et al. [5] performed accuracy tests in 1999, on a former version of the VICON system (VICON 370) with only 6 cameras and found errors on a fixed 50mm distance of 0.062mm [0.183mm] (RMS [max]), of 0.129mm [0.557mm] on a 9cm distance between two markers on a moving plate and an error in angles of 1.4° [4.6°].

Moreover, Gorton et al [6] explored in 2009 the source of variability of human motion capture measurements by comparing 12 laboratories. The average instrumental error on a known distance was 1.2mm across labs with an average between lab deviation of only 0.9mm. When comparing instrumental errors to marker placement differences, they concluded that “marker placement differences between examiners are shown to be the most likely source of between site variability”.

Thus, since marker placement variability is way higher than between system variability, we believe that the difference between systems is negligible our case since the measurements were performed at the same lab with the same markerset and palpation method between datasets.

A specific section was added to the limitation section of the discussion:

L371: “Secondly, the cohort combined datasets assessed with different motion capture systems brand and generations. A recent study from 2020 by Topley et al. [4] showed that modern systems were equivalent in terms of instrumental errors and Gorton et al. [6] showed that the instrumental error was lower than marker misplacement errors when assessing variability. Thus, since both databases were measured in the same laboratory, with the same number of cameras, with the same marker set and the same palpation techniques for marker placement, we assume that the instrumental errors between systems did not impact the results of the study.”

Data Classification
• Page 8, line 162: The authors stated that “the optimal number of clusters was identified with the silhouette criterion.” Please specify and add a reference.

The silhouette criterion was introduced by Rousseeuw in 1987 [13] and is a measure of how well a point belong to its cluster when compared to the closest cluster. As Rousseeuw phrases it, when the silhouette is close to 1, this means that “the within [cluster] dissimilarity is much smaller than the smallest between [cluster] dissimilarity”. By measuring the silhouette criterion for all points it is possible to have a measure of how well clusters are defined and choose the most appropriate number of clusters.

The reference and a more detailed description were added to the manuscript:

L174: “The silhouette criterion [13] was used to select the number of clusters. This widely used tool represents a measure of how well an object belongs to its cluster when compared to the closest cluster. By averaging the silhouette criterion of all objects, it is possible to have an estimation of how well the clusters are separated. The criterion was computed for 2 to 10 clusters and the number with the highest silhouette was selected as optimal and used in the study.”

Data Analysis
• Page 9, lines 171-172: The authors stated that “the Chi2 test was used for proportions (satisfied or not, side of surgery, bilateral knee OA, sex).” Please clarify, if patients with bilateral knee OA were included as well!? Up to this point, I thought that only unilateral patients had been included. Please discuss the potential effect of including bilateral affected patients besides unilateral affected patients! Even if the difference in the distribution of unilateral and bilateral patients is not different between the groups, in my opinion, it would have been better to include only unilaterally affected patients in the analysis.

In our opinion, it would be more representative of the patient population to keep both included. Indeed, in a study on prevalence of generalized osteoarthritis in knee OA patients, Forestier et al. [10] found 66% of patients with bilateral knee OA and 52% of patients with at least one out of the three definitions of generalised OA in the studied population of 305 patients. At the first steps of the study, we thought it would be interesting to see whether groups of unilateral and bilateral appear in the automatic classification. This point was added to the initial hypotheses.

L97: “We hypothesize that multiple clusters will emerge with a higher functional group, a lower functional group including mostly patients with bilateral knee OA, and a limping group with upper body gait deviations”

The method was updated to clarify, L104: “One hundred patients planned for unilateral primary TKA due to end-stage knee OA were included in this study. Thirty-nine patients had bilateral knee OA.”

The discussion was updated as follow,

L345: “Contrary to our hypothesis, there was no differences in terms of unilateral or bilateral knee affection between the three clusters.”

3 RESULTS
Characteristics of clusters before surgery
• Table 1: p-values for the sex distribution are missing. Please also add the superior p-values for KW or CHI2 and not only the p-values for the pairwise comparison.

For the sake of clarity, we only reported p-values below the threshold of 0.05 in this table and all results in supporting information. No p-value means the outcome of the statistical test was negative. This is now specified in the table description. We prefer this solution, however, if the reviewer prefers, we can add all p-values in the table.

L213: “*Only the p-values below 0.05 were reported for clarity.*”

4 DISCUSSION
• Significant differences in walking speed exists between patient clusters (2 vs. 3 and 3 vs. 1) and compared to the control group (all clusters). Please discuss the potential effect of the difference in walking speed on the results of the study and conclusions made.

Indeed, cluster 3 is slower than clusters 1 and 2 and differences in terms of walking will impact those differences. However, Fukuchi et al. [14] showed that gait speed impacted mostly the amplitude of the considered gait parameters, either kinematics or kinetics. This is consistent with the reduced RoM of knee and ankle in the sagittal plane for cluster 3 compared to clusters 1 and 2. However, we see that for this cluster, the kinematic patterns of the thorax and pelvis are modified and indicate a limping behaviour that does not seem to be only due to the limited walking speed.

This was added to the discussion:
L268: “CL3 had slower gait speed than all other groups, however, as gait speed impacts mostly the amplitude of the gait parameters and not their pattern [14], this deviation of pattern is likely typical of this population.”

**Reviewer #2:** In this study, the authors utilize an unsupervised clustering method to identify mechanisms that candidates for total knee arthroplasty (TKA) may utilize to compensate for pain or lack of function and to determine whether these mechanisms persist after TKA. To this end, the authors analyze a series of whole-body kinematic parameters (excluding the knee) and spatiotemporal parameters of gait. The authors then compare these groups among them and to a control group as a function of their patient reported outcomes. The authors identify three phenotypes from the TKA patients. Identifying different phenotypes of TKA patients is a crucial step to understand how the surgery can be tailored to these groups to improve success and satisfaction. In this way, the manuscript is interesting, because it investigates how other joints may compensate for lack of optimal knee function. However, I have some concerns with the study in its current form, which dampen my enthusiasm for the presented work. Some comments are, in order:
The goal of the manuscript is to identify clusters of TKA candidates by their compensatory mechanisms. The authors identify three groups according to their strategy to walk. However, how can the authors be sure that these are compensatory mechanisms? Could it be that these people just walked in a particular manner but that it is not a compensatory mechanism?

We thank the reviewers for these interesting questions and remarks. Regarding the compensatory mechanism, we may have misused this term. Indeed, since their identification would require additional clinical data that we don’t have at our disposal in this database, it was chosen to only consider “gait deviations” in the paper. In this way, the objective stated fits better the design of the study. Thus, the term “compensation” was replaced by “gait deviation” in the title and throughout the manuscript.

In this way, the authors compare the groups to the control group; however, it is unclear whether clusters also exist in healthy patients and whether any of those clusters is similar to the TKA clusters.

Regarding the presence of similar clusters in the general elderly population, the present size of the control group was too limited to perform clustering on it, thus we don’t know if the clusters identified in the OA patients exist in the control population.

This is a relevant research question; however, this would require additional research that is not in the scope of this study. We believe that the fact the clusters may be in the healthy population does not impact the clinical findings of the paper stating that: patients with high functional levels may not improve their function but will improve their pain levels while patients with low levels of function are likely to improve both function and pain. This finding will be useful for surgeons during the information to the patients and specifically to manage patient’s expectations.

On a similar note, recent studies on knee functional phenotyping such as the popular CPAK (Coronal Plane Alignment of the Knee) [15] showed similar phenotypes between healthy population and OA population. However, this tool is still relevant in planning surgery and understanding the patient’s profile.

The authors hypothesize that three groups would appear: high functional, low functional, limping group. However, the authors do not provide any criteria for which criteria will be used to assess function (i.e., is it a particular gait or spatiotemporal parameter or is it a compound of various metrics?) or any criteria for establishing whether a patient has high or low function. Without these criteria, it is unclear that the hypothesis can be tested.

This hypothesis was based on a previous study by Young-Shand that mentioned high and low function groups in a population of patients undergoing TKA. The criteria used was simply the amount of deviation with respect to the control group, especially in terms of gait speed, a key indicator of functional levels [1-3]. However, we did not set thresholds for high or low functional levels. To phrase this appreciation in a relative manner, we are now talking about a “higher” and a “lower” functional group throughout the manuscript.

This was specified in the introduction:
L97: “We hypothesize that multiple clusters will emerge with a higher functional group, a lower functional group, and a limping group with upper body gait deviations. The level of function will be assessed by comparison to with the control group in terms of walking speed, gait kinematics, and spatio-temporal parameters.”

More importantly, the authors do not present statistical analysis that confirms that the three obtained groups have different kinematics and spatiotemporal parameters of gait. In this way, it is unclear from the results whether the hypothesis can be confirmed or rejected.

Regarding the lack of statistical analysis, we disagree with this point. We performed multiple statistical analyses described between L185 and L193 in the “Data Analysis” section in the results, in table 1 and under visual representation in figures 1, 2 and 3. All results were not reported in the text to make the paper easier to read and understand. However, all tests were reported in the supporting information 1, 2, 3 and 4. We did notice a typo in supporting information 4 so we updated a new one with this revision.

In this study, the clusters of patients were compared between each other before surgery, to the control group before and after surgery and finally their pre-surgery visit was compared to their post-surgery one to assess their evolution. The outcomes compared were the clinical data (sex, age, height, BMI, % bilateral, WOMAC, SF12), the kinematic features of the thorax, pelvis, hip, knee, ankle, and foot progression as well as the spatio-temporal parameters including walking speed, stride time, stride length, cadence, step time, step length, step width, time of foot off, percentage of single support and percentage of double support.

As explained in the section Data Analysis:
L185: “After data clustering, the clusters pre-surgery were compared to each other in terms of clinical data, kinematics features, and spatio-temporal data. The analysis was blind to the type of surgery or implant. The Kruskall-Wallis test with post-hoc Wilcoxon tests was used to compare the multiple clusters for all continuous data. The Chi2 test was used for proportions (satisfied or not, side of surgery, bilateral knee OA, sex). The evolution of each cluster was assessed by comparing the clinical data, kinematics features, and spatio-temporal data measured pre-surgery to the ones measured one year after surgery with Wilcoxon tests. Finally, each cluster was compared before and after surgery to the control group with a Wilcoxon test. Statistical significance was set at p < 0.05.”

I find the way the results and discussion are presented confused. The authors compare groups among themselves at the same time that they compare them to the control group.

We understand that the results can be a bit confusing due to the large number of tests and outcomes which is why we chose to present mostly the results graphically with all details in the supporting information. To perform a complete analysis, we performed statistical tests between clusters pre-surgery, between visits (pre-surgery and 1-year post-surgery) for the three clusters, and the visits of each cluster were also compared to the control group.

For example, in lines 256-258, it is unclear whether the TKA clusters were different in their pain and step width or was just CL1 compared to healthy.

As stated in the method, all groups were compared to the control group. This sentence of the discussion was slightly clarified:

L283: “CL1 (n=59) and CL2 (n=20) were similar in terms of patient characteristics (i.e. age, sex, BMI) and spatio-temporal parameters, but CL1 presented significantly less pain before surgery than CL2 and larger step width than the control group.”

To achieve their goal of identifying patterns of full body compensation, I would argue that the authors have to show that differences exist in the kinematics across TKA patients (other than the knee), that these differences are caused by compensatory mechanisms, how these differences relate to knee kinematics, and how these differences affect outcomes (i.e., PROMS). I believe most of this is in the manuscript, but it is not presented in a manner that helps identify/highlight these differences, making it hard for the reader to understand the message of the manuscript. I suggest re-structuring the results to present the clusters, discussing their demographics. Then, present their differences in compensatory mechanisms (i.e., why were they identified as different groups?). Then present their differences in knee kinematics and relate these to the control group. Finally, discuss their differences in PROMs and relate these to surgical technique or implant/limb alignment.

We are not sure to fully understand how these suggestions would differ from the existing structure of the paper. Indeed the steps suggested by the reviewer were done and presented in the results (L196 to L251):

- 1^st^ paragraph describes the number of clusters and demographics
- 2nd paragraph presents differences pre-surgery in terms of joint & segment kinematics
- 3rd paragraph presents differences pre-surgery in terms of spatiotemporal parameters
- 4th paragraph presents evolution of PROMs at one-year post-surgery
- 5^th^ paragraph presents evolution of kinematics and spatiotemporal parameters one-year post-surgery

Other comments/questions:
How was lack of knee OA assessed in the healthy group?

We checked the inclusion-exclusion criteria mentioned in the manuscript (L106 to L112) during an interview before the measurement to check whether participants had limitations during gait, especially due to knee pain or whether they had a previous diagnostic of knee OA. There were no X-rays performed to avoid exposing controls to unnecessary radiations. Thus, some participants of the control group may have had asymptomatic knee OA, to clarify, we added “symptomatic” to knee OA:

L109: “The healthy participants were matched in age and gender with the patient population and were included in the absence of symptomatic knee OA or other orthopaedic or neurologic problems potentially affecting gait or balance”.

Were there differences in surgical technique or implant position among patients in the different clusters?

Despite differences in type of implant between the two datasets, the percentage of patients from each dataset was the same in each clusters as in the cohort and, there was no differences in terms of implant types between the clusters.

We added the results of these tests to the main body of the paper;

Results – L205: “There was no significant difference in proportion of patients from each dataset in each cluster, respectively 15.3%, 30% and 29% of patients from cohort 2 for CL1, CL2 and CL3 and with the porporition for the whole cohort (21%). There were differences in terms of implant type between datasets but there was no differences between clusters (Table 2).”

Discussion – L378: “Third, the cohorts presented differences in terms of implant which was mostly due to the evolution of the implant market. However, the percentage of patients from each dataset was the same in each clusters as in the cohort and, there was no differences in terms of implant types between the clusters. This suggests that the post-surgery differences between clusters may be due to the pre-surgery functional profiles. The fact that the clustering was performed on pre-surgery data may have helped tempering the implants’ influence.”

L105 – did the participants had any other joint affected by advanced OA, which may have contributed to their affected gait? Was this different across groups?

Multiple patients had bilateral knee OA but no other pathologies of the lower limb and/or spine. There were no differences between groups in terms of unilateral/bilateral knee affection percentage.

L 110 – what were these inclusion/exclusion criteria?

These inclusion/exclusion criteria are the ones mentioned in the paragraph before L106 to L112.

L117 – can the authors include a brief description of the surgical procedure? While the authors provide a reference, the

Additional details on the surgery were added in the manuscript:
L121 : “The surgery were done routinely by a medial parapatellar approach, with use of fully cemented implants. For the first dataset, most of the patients had a tibia first technique, using a mechanical alignment. A fixed bearing was used in all patients but in the case of ultra-congruent (UC) constraint. In the second dataset, a kinematic alignment was preferred when using TKA with a medial pivot polyethylene, with a femur first, caliper-measurement technique. Cementing technique has been always realised in a two-step manner, by means of antibiotic loaded PMMA (Palacos). Patella resurfacing has been carried out routinely for the second dataset, independently from the constraint used, given a tendency towards too many revision for anterior knee pain.”

The analysis was performed blindly to the type of implant or surgery. This was added to the manuscript.

L186: “The analysis was blind to the type of surgery or implant.”

L162 – can the authors briefly describe the silhouette criterion?

The silhouette criterion was introduced by Rousseeuw in 1987 [13] and is a measure of how well a point of belong to its cluster when compared to the closest cluster. As Rousseeuw phrases it, when the silhouette is close to 1, this means that “the within [cluster] dissimilarity is much smaller than the smallest between [cluster] dissimilarity”. By measuring the silhouette criterion for all points it is possible to have a measure of how well clusters are defined and choose the most appropriate number of clusters.

The reference and a more detailed description were added to the manuscript:

L174: “The silhouette criterion [13] was used to select the number of clusters. This widely used tool represents a measure of how well an object belongs to its cluster when compared to the closest cluster. By averaging the silhouette criterion of all objects, it is possible to have an estimation of how well the clusters are separated. The criterion was computed for 2 to 10 clusters and the number with the highest silhouette was selected as optimal and used in the study.”

L184-185 – The authors indicate in the methods that the control group was matched by age/sex/bmi to the TKA patients. How is it possible that one of the clusters is significantly older than the controls?

The whole patient group was similar as shown in table 1. However, when selecting a subgroup, it is possible to be on one end or the other of the age distribution, the mean of this subgroup will be different than the mean of the whole distribution. The cluster 3 regrouped patients with the highest gait limitations which is consistent with older age.

L200-206 –can the authors be more specific as to the direction of the deviations?

The authors tried to stay general to avoid lengthy descriptions that may be difficult to read. Due to the large amount of data and results, we tried to keep the results as graphic as possible to give a good overview of the phenomenon at stake. The detailed information are present in the supporting information. The paragraph was slightly modified as follows to provide more details:

L225: “Regarding joint and segment kinematics pre-surgery, CL1 was mainly characterized by varus knee and sagittal plane pelvis and hip deviations compared to the control group (Figure 1, Supporting Information 2). CL2 was characterized by gait deviations of the thorax, pelvis, and knee sagittal plane kinematics (Figure 1, Supporting Information 2). CL3 was characterized by deviations of the thorax and pelvis in the frontal and transverse plane including increased RoM (e.g. thorax frontal) and modified patterns (pelvis frontal) as well as the external foot progression angles and reduced ranges and peak values of the hip, knee and ankle in the sagittal plane (Figure 1, Supporting Information 2).”

L217-225 – PROMs are one of the main outcomes evaluated and used in the discussion; however, they are included in a supplement and not in the main manuscript. Can the authors please include the PROMs scores and their changes and whether these changes reached the MCID?

We chose a graphical representation to ease the reading of the paper. All numbers are indeed reported in supporting information, which are part of the paper. Including all those tables in the main body would make the paper overly cumbersome to read.

Regarding the MCID, since there are multiple methods of computation [16], no real consensus yet for the MCID of PROMs in orthopaedic [17, 18], and the PROMs a only secondary outcomes of this study, mostly used to describe the population in the cluster, we will let it to the reader discretion to conclude on this question. Moreover, post-surgery evolution of PROMs was largely described in the literature with wider cohorts in registry-based studies.

L276 – how did the authors conclude that the pattern is “safe”? What makes a pattern “safe”?

The term “safe” was used in the paper cited in the same sentence [19]. Gouelle et al. consider that widening the base of support will increase balance and thus indicate that the patient prefers “safe” walking pattern. We put it between guillemet to mark the fact that we cited this paper and the fact that this was formulated in Layman’s term.

L258-261 – can the authors quantify how “close” the parameters were to the control group? Perhaps determining whether they were statistically significant/clinically meaningful or not.

We are not sure to fully understand the question, all feature values for the patient population, clusters before/after surgery, and control group, as well as all statistical tests, were reported as results and in the supporting information to quantify this “closeness”. Since this part is the discussion, we do not think that it is necessary to mention again all those values here to avoid redundancy. We prefer to speak in general terms with an overview of the results to help their understanding.
The lack of MCID to assess clinically meaningful differences was a choice discussed in the previous paragraphs of the answer to the reviewer.

L262 – can the authors describe the improvements in WOMAC? Were these above MCID?

The evolution of WOMAC scores was described in figure 1 and the exact values were reported in supporting information. As reported by MacKay et al. [20], “although the WOMAC is a commonly used measure for a single condition, the variability in methods used to calculate MCID and PASS estimates results in a range of values across studies making it unclear whether values reported in the literature can be applied with confidence.” Thus, since the WOMAC is only a secondary outcome, mostly used to characterise the groups as is commonly done in TKA studies, we will not go in depth in analysing improvements of WOMAC and the multiple MCID reported in the literature.

1.	Fritz S, Lusardi M. White paper:“walking speed: the sixth vital sign”. Journal of geriatric physical therapy. 2009;32(2):2-5.

2.	Lusardi MM. Is Walking Speed a Vital Sign? Absolutely! Topics in Geriatric Rehabilitation. 2012;28(2).

3.	Middleton A, Fritz SL, Lusardi M. Walking Speed: The Functional Vital Sign. Journal of Aging and Physical Activity. 2015;23(2):314-22. doi: 10.1123/japa.2013-0236.

4.	Topley M, Richards JG. A comparison of currently available optoelectronic motion capture systems. Journal of Biomechanics. 2020;106:109820. doi: <https://doi.org/10.1016/j.jbiomech.2020.109820>.

5.	Richards JG. The measurement of human motion: A comparison of commercially available systems. Human Movement Science. 1999;18(5):589-602. doi: <https://doi.org/10.1016/S0167-9457(99)00023-8>.

6.	Gorton GE, Hebert DA, Gannotti ME. Assessment of the kinematic variability among 12 motion analysis laboratories. Gait and Posture. 2009;29(3):398-402. doi: 10.1016/j.gaitpost.2008.10.060.

7.	Bonnefoy-Mazure A, Armand S, Sagawa Y, Suva D, Miozzari H, Turcot K. Knee Kinematic and Clinical Outcomes Evolution Before, 3 Months, and 1 Year After Total Knee Arthroplasty. J Arthroplasty. 2017;32(3):793-800. doi: 10.1016/j.arth.2016.03.050.

8.	Messier SP, Beavers DP, Herman C, Hunter DJ, DeVita P. Are unilateral and bilateral knee osteoarthritis patients unique subsets of knee osteoarthritis? A biomechanical perspective. Osteoarthritis and Cartilage. 2016;24(5):807-13. doi: <https://doi.org/10.1016/j.joca.2015.12.005>.

9.	Creaby MW, Bennell KL, Hunt MA. Gait Differs Between Unilateral and Bilateral Knee Osteoarthritis. Archives of Physical Medicine and Rehabilitation. 2012;93(5):822-7. doi: <https://doi.org/10.1016/j.apmr.2011.11.029>.

10.	Forestier R, Francon A, Briole V, Genty C, Chevalier X, Richette P. Prevalence of generalized osteoarthritis in a population with knee osteoarthritis. Joint Bone Spine. 2011;78(3):275-8. doi: <https://doi.org/10.1016/j.jbspin.2010.09.024>.

11.	Favre J, Jolles BM. Gait analysis of patients with knee osteoarthritis highlights a pathological mechanical pathway and provides a basis for therapeutic interventions. EFORT open reviews. 2016;1(10):368-74.

12.	Baker R, Leboeuf F, Reay J, Sangeux M. The Conventional Gait Model - Success and Limitations. In: Müller B, Wolf SI, editors. Handbook of Human Motion: Springer International Publishing AG; 2017. p. 1-19.

13.	Rousseeuw PJ. Silhouettes: A graphical aid to the interpretation and validation of cluster analysis. Journal of Computational and Applied Mathematics. 1987;20:53-65. doi: <https://doi.org/10.1016/0377-0427(87)90125-7>.

14.	Fukuchi CA, Fukuchi RK, Duarte M. Effects of walking speed on gait biomechanics in healthy participants: a systematic review and meta-analysis. Systematic reviews. 2019;8:1-11.

15.	MacDessi SJ, Griffiths-Jones W, Harris IA, Bellemans J, Chen DB. Coronal Plane Alignment of the Knee (CPAK) classification. Bone Joint J. 2021;103-b(2):329-37. doi: 10.1302/0301-620x.103b2.Bjj-2020-1050.R1. PubMed PMID: 33517740; PubMed Central PMCID: PMCPMC7954147.

16.	Molino J, Harrington J, Racine-Avila J, Aaron R. Deconstructing the Minimum Clinically Important Difference (MCID). Orthopedic Research and Reviews. 2022;14(null):35-42. doi: 10.2147/ORR.S349268.

17.	Bloom DA, Kaplan DJ, Mojica E, Strauss EJ, Gonzalez-Lomas G, Campbell KA, et al. The Minimal Clinically Important Difference: A Review of Clinical Significance. The American Journal of Sports Medicine. 2023;51(2):520-4. doi: 10.1177/03635465211053869. PubMed PMID: 34854345.

18.	Copay AG, Eyberg B, Chung AS, Zurcher KS, Chutkan N, Spangehl MJ. Minimum Clinically Important Difference: Current Trends in the Orthopaedic Literature, Part II: Lower Extremity: A Systematic Review. JBJS Reviews. 2018;6(9):e2. doi: 10.2106/jbjs.Rvw.17.00160. PubMed PMID: 01874474-201809000-00003.

19.	Gouelle A, Mégrot F, Müller B. Interpreting spatiotemporal parameters, symmetry, and variability in clinical gait analysis. Handbook of Human Motion, eds B Müller, SI Wolf, GP Brueggemann, Z Deng, A McIntosh, F Miller, and WS Selbie (Cham: Springer International Publishing). 2018;689:707.

20.	MacKay C, Clements N, Wong R, Davis AM. A systematic review of estimates of the minimal clinically important difference and patient acceptable symptom state of the Western Ontario and McMaster Universities Osteoarthritis Index in patients who underwent total hip and total knee replacement. Osteoarthritis and Cartilage. 2019;27(10):1408-19. doi: <https://doi.org/10.1016/j.joca.2019.05.002>.
